# Supercritical CO_2_-Assisted Spray Drying of Strawberry-Like Gold-Coated Magnetite Nanocomposites in Chitosan Powders for Inhalation

**DOI:** 10.3390/ma10010074

**Published:** 2017-01-18

**Authors:** Marta C. Silva, Ana Sofia Silva, Javier Fernandez-Lodeiro, Teresa Casimiro, Carlos Lodeiro, Ana Aguiar-Ricardo

**Affiliations:** 1 LAQV-REQUIMTE, Departamento de Química, Faculdade de Ciências e Tecnologia, Universidade NOVA de Lisboa, Campus de Caparica, Caparica 2829-516, Portugal; martasilva686@gmail.com (M.C.S.); asm.silva@campus.fct.unl.pt (A.S.S.); teresa.casimiro@fct.unl.pt (T.C.); 2 BIOSCOPE Research Group, UCIBIO@REQUIMTE, Chemistry Department, Faculty of Science and Technology, University NOVA of Lisbon, Caparica Campus, Caparica 2829-516, Portugal; j.lodeiro@fct.unl.pt; 3 CICS-UBI, Health Sciences Research Center, Faculdade de Ciências da Saúde, Universidade da Beira Interior, Av. Infante D. Henrique, Covilhã 6200-506, Portugal; 4 PROTEOMASS Scientific Society, Rua dos Inventores, Madam Parque, Caparica Campus, Caparica 2829-516, Portugal

**Keywords:** lung diseases, dry powders, magnetic nanoparticles, nanocomposites, SASD, pulmonary delivery

## Abstract

Lung cancer is one of the leading causes of death worldwide. Therefore, it is of extreme importance to develop new systems that can deliver anticancer drugs into the site of action when initiating a treatment. Recently, the use of nanotechnology and particle engineering has enabled the development of new drug delivery platforms for pulmonary delivery. In this work, POXylated strawberry-like gold-coated magnetite nanocomposites and ibuprofen (IBP) were encapsulated into a chitosan matrix using Supercritical Assisted Spray Drying (SASD). The dry powder formulations showed adequate morphology and aerodynamic performances (fine particle fraction 48%–55% and aerodynamic diameter of 2.6–2.8 µm) for deep lung deposition through the pulmonary route. Moreover, the release kinetics of IBP was also investigated showing a faster release of the drug at pH 6.8, the pH of lung cancer. POXylated strawberry-like gold-coated magnetite nanocomposites proved to have suitable sizes for cellular internalization and their fluorescent capabilities enable their future use in in vitro cell based assays. As a proof-of-concept, the reported results show that these nano-in-micro formulations could be potential drug vehicles for pulmonary administration.

## 1. Introduction

Lung cancer is one of the most common and leading causes of cancer death worldwide [1,2]. To address this problem, different systems that can easily carry active drugs into the site of action and thus initiate the respective cancer treatments have been widely investigated [3]. A suitable drug carrier, or drug delivery system (DDS), should protect the drug against degradation until it reaches the desired site of action [4]. Therefore, the treatment efficacy mostly depends on the system by which the therapeutic dose of the drug is delivered (carrier and administration route) [5,6].

As local administration to the lungs has become one of the best alternatives to improve lung cancer outcomes [6], pulmonary delivery has been widely investigated as a primary route of administration, as it enables direct targeting to the lungs for both local and systemic treatment [7]. Pulmonary delivery together with controlled release systems allows drug protection from rapid degradation or clearance; it enhances the accumulation in the cells by increasing its bioavailability, therefore lowering the amount of drug required, reducing harmful side effects and reducing costs [8].

Recently, nanotechnology has been combined with pulmonary delivery systems, and nanoparticles (NPs) can be used as efficient drug carriers that provide a controlled release of the drug and accurately recognize different cellular types [9,10,11]. In fact, over the last two decades, nanotechnology has provided extraordinary outcomes in both drug delivery and cell targeting [3,12]. Thus, the merging of nanotechnology, particle engineering, cancer biology, and pulmonary delivery can bring new improvements for the diagnosis and therapy of lung cancer [6,13]. Due to their small size (1–150 nm), NPs can easily penetrate the deep tissue and be taken up by cells more quickly while protecting the drugs at both extracellular and intracellular levels [14,15]. These achievements have been shown to improve drug half-lives and retention, which will substantially decrease unwanted side effects. Besides their therapeutic capabilities, nanoparticles can also be designed to exhibit diagnostic features, thus creating theragnostic nanosystems. Nanoparticles with theragnostic capabilities can offer many benefits for lung disease patients including real-time diagnosis, adequate therapy for individual patients (personalized medicine), and reduction of adverse drug effects [6,13].

In an attempt to produce theragnosis systems, different types of nanoparticles have been investigated: (i) metal (such as gold (Au), ferric (Fe), and silver (Ag)) [16]; (ii) polymeric, (such as polyethylene-glycol (PEG) [17], silica (Si) [18], and poly(lactic-co-glycolic acid) (PLGA)) [19]. In fact, metallic NPs have proven to be great drug carriers and contrast agents in cancer treatment [16]. Also, they are readily functionalized, which is a matter of extreme importance since NPs lacking functionalization are more likely to interact with plasma proteins or be uptaken by macrophages, and can be used as active targeting nanocarriers to target specific cellular types. By controlling nanoparticles’ surface properties, a stealth character can be conferred to the nanosystems, preventing agglomeration and opsonization, therefore protecting them and thus increasing their residence time and ensuring the delivery of the drug to the desired site of action [20,21,22].

Magnetite NPs have been studied for several applications, in particular for medical diagnosis due to their magnetic properties by using MRI (magnetic resonance imaging) therapy and targeted drug delivery. These nanoparticles also show superparamagnetic behavior when working above their blocking temperature, which allows them a fast response to applied magnetic fields. However, in the majority of cases, naked particles tend to be very unstable over long periods of time and lean towards agglomeration [23,24]. Moreover, although they show low toxicity, they can also cause cell damage by forming reactive oxygen species. Some strategies comprising grafting or coating are used to prevent this toxicity and the possible aggregation [25].

Gold nanoparticles (AuNPs) have many advantages due to their inertness, non-toxic core, and excellent metal stability. Also, AuNPs are readily functionalized, biocompatible, have high light absorption, and are highly stable. Concerning these significant advantages, this work is focused on the synthesis of gold-coated magnetic nanocomposites as gold shells able to confer stability to the magnetic core, functionality, and biocompatibility properties. Also, the magnetic core will confer to the NPs magnetism that is capable of being used in magnetic resonance imaging and hyperthermia [23]. POXylation (grafting with oligo(2-ethyl-oxazolines), a similar strategy to PEGylation, currently under investigation to circumvent PEG hepatoxicity issues, has also been employed. Besides the ability to protect nanoparticles, POXylation also add fluorescent capabilities to the nanoparticles enabling an in vitro real time analysis of particles’ course within cell environments [26,27,28]. However, nanoparticles are not in a suitable size for deep lung deposition, as they can be easily exhaled or muccociliary cleared out [29]. It has been established that the optimal aerodynamic diameter for particle deposition into the deep lung region is between 0.5 and 5 μm [30,31,32]. Therefore, to overcome this limitation, dry powder formulations containing both nanoparticles presented as nanocomposites and micro particles (nano-in-micro formulations) are the focus of study. These dry powder formulations make use of excipients capable of carrying the nanoparticles into the site of action. 

Chitosan (CHT), as a biodegradable, non-toxic [33,34,35], mucoadhesive, and biocompatible polymer, has been chosen by many researchers as a microcarrier for therapeutic agent delivery through the pulmonary route [32,33,36,37]. Herein, CHT is used as a microcarrier for the POXylated strawberry-like gold-coated magnetite nanocomposites [20]. The encapsulation process is performed using a sustainable technology, SASD, that makes use of Supercritical Carbon Dioxide (scCO_2_) which is mixed and solubilized in the liquid solution (thus minimizing the use of organic and not so environmentally friendly solvents) and further expansion of this mixture through a nozzle, a process patented by E. Reverchon (patent US 7,276,190 B2) [32,38,39]. This process will allow us to obtain particles with smaller sizes than conventional techniques such as spray-drying or spray freeze-drying, among others [40].

**Figure 1:**
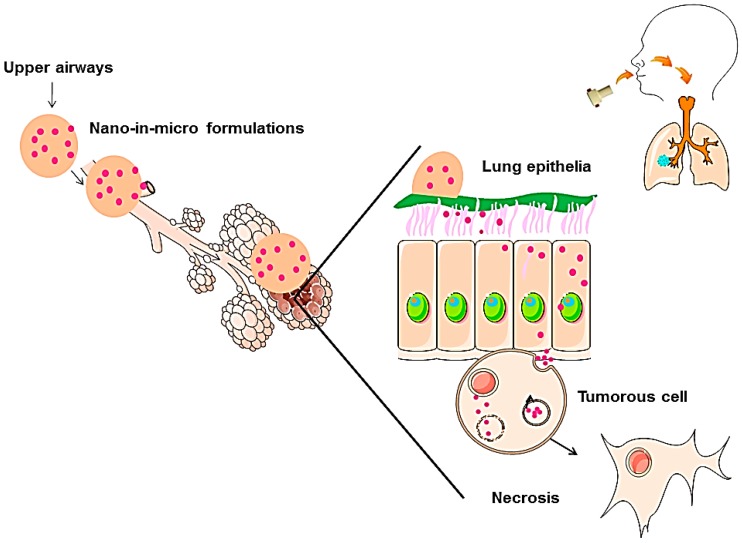
Mechanism of nanoparticle delivery through an aerosol of nano-in-micro formulations. Adapted from A. Silva et al. [20].

This work is focused on the development of new engineered POxylated strawberry-like partially gold-coated magnetite nanocomposites micronized into CHT [20,39,41], to produce dry powders suitable for deep lung deposition [32]. This mechanism is represented in Figure 1. Additionally, IBP was encapsulated to evaluate if this system can be used as a synergetic platform [27,32]. The final nano-in-micro drug delivery system is herein studied as a proof-of-concept and therefore, in order to understand the effectiveness of this system, a more detailed study is being taken by our group to prove its viability.

## 2. Results and Discussion

### 2.1. Nanoparticle Characterization

Size measurements of the strawberry magnetic@gold composites were obtained by TEM images, and Zeta Potential. Both data were reported previously [25].

**Figure 2:**
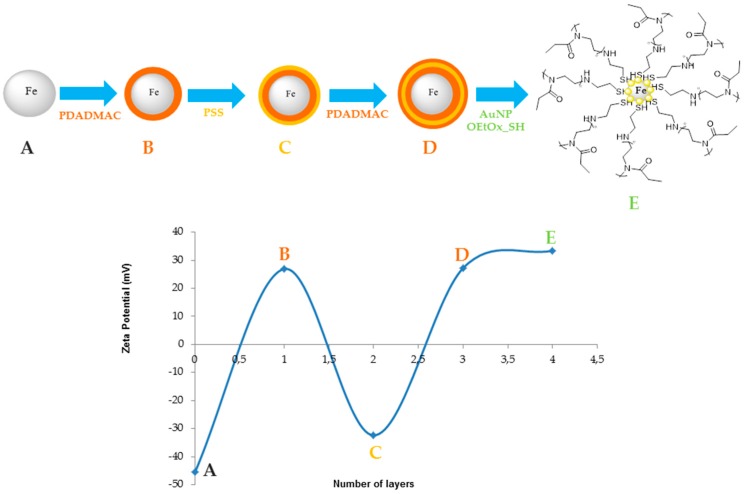
Zeta potential of the different layers, where the fourth layer corresponds to the final nanoparticles. PDADMAC—poly-diallyldimethylammonium chloride; PSS—poly-sodium 4-tyrenesulfonate.

Magnetic@gold nanocomposites were synthesized using a layer-by-layer deposition by adding three different layers of two polymers (one negative and one positive) onto the surface of the magnetic core, and then the partial gold shell was made in situ onto the magnetic nanoparticle. As depicted in Figure 2, the deposition of poly-diallyldimethylammonium chloride (PDADMAC) was clearly demonstrated through a complete reversal of surface charge, from negative to positive. As a layer of poly-sodium 4-styrenesulfonate (PSS) was deposited onto the particles, zeta potential was again reversed (to negative), corroborating this new layer deposition. As the process of PDADMAC deposition was repeated, the last particles exhibiting three polymeric layers showed a positive surface charge. The addition of a gold shell coated with the fluorescent oligo(2-ethyl-2-oxazoline) showed that the zeta potential of the particles had maintained its positive value of approximately 33.6 mV.

**Figure 3:**
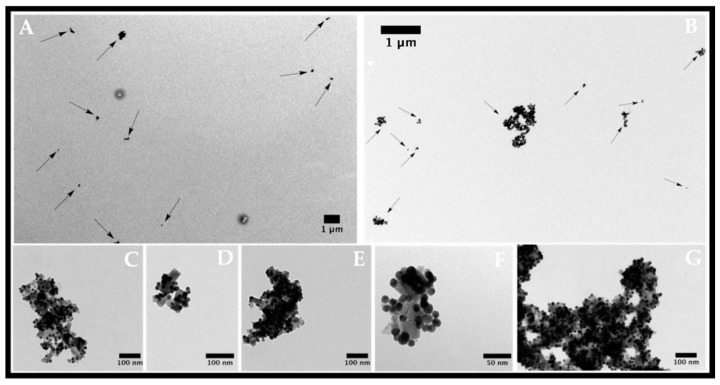
Transmission electron micrographs of strawberry Fe/Au nanomaterials functionalized with oligo(2-ethyl-2-oxazoline) in different magnifications. (**A**,**B**) magnetite nanoparticles; (**C**–**G**) Strawberry-like gold magnetite nanocomposites functionalized with oligo(2-ethyl-2-oxazoline).

The positive zeta potential exhibited at the surface of these new types of nanocomposites is expected to increase cellular uptake due to their electrostatic interaction with the negatively charged phospholipidic membrane. Their sizes are well suited for delivery into tumor cells. TEM images (Figure 3) show that all gold nanoparticles are anchored to the magnetic surfaces of magnetite nanoparticles, therefore forming stable nanocomposites. Nanocomposites with a diameter ranging within 50–200 nm were then obtained, which had a suitable size for cellular internalization. These nanocomposites have already been tested in a previous work applied to Proteomics, and have presented excellent stability [25].

**Figure 4:**
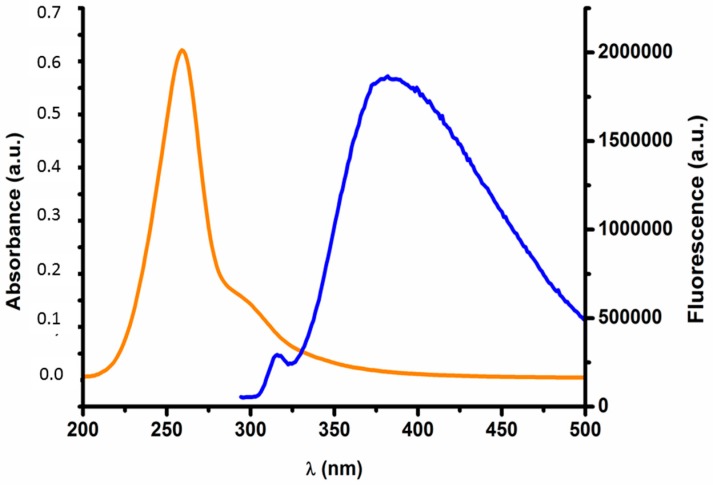
Fluorescence vs. Absorbance of the functionalized nanoparticles. The emission band can be denoted at 384 nm (blue line) and the absorption band of the polymer can be seen between 240–320 nm (orange line).

Since magnetite gold coated nanoparticles do not present any intrinsic fluorescence, oligo(2-ethyl-2-oxazoline) end terminated with cysteamine (POx-SH) was conjugated to the nanoparticles in order to produce systems able to be tracked within cell environments using fluorescent microscopy assays. In fact, UV-Vis and fluorescent assays confirmed the grafting of POx-SH to the surface of the nanoparticles. Figure 4 shows both the emission and excitation spectra of POxylated nanoparticles. The representative bands of POx-SH are maintained after particle grafting (λ_exc_ = 300; λ_emm_ = 384 nm) [26,27,42].

### 2.2. Characterization of Nano-in-Micro Formulations 

#### 2.2.1. Morphological and Physical-Chemical Properties

**Figure 5:**
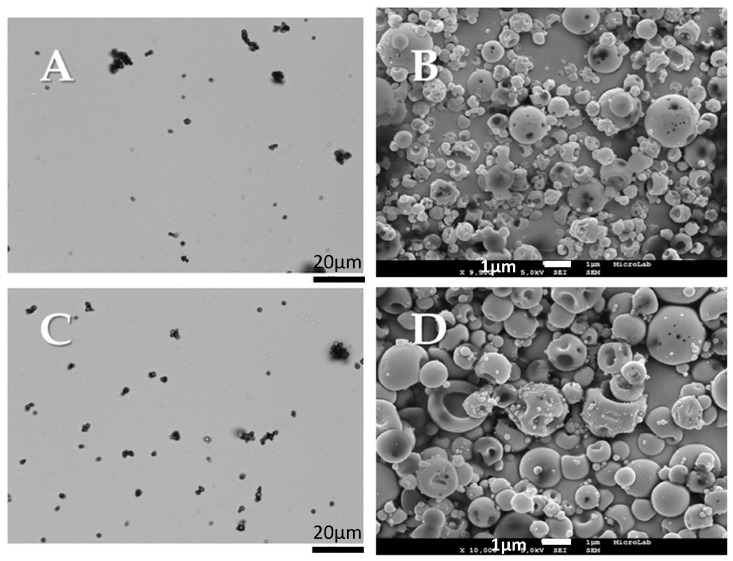
Morphological analysis of the produced particles: CHT: (**A**) Morphologi G3 image; (**B**) SEM image; CHT_Fe@Au_POX-SH: (**C**) Morphologi G3 image; (**D**) SEM image.

The morphological analysis of the particles was performed by scanning electron microscopy (SEM) and using Morphologi G3 equipment, where a population of 30,000 particles was analyzed. The micrographs displaying the shape of the particles are presented in Figure 5, and the results obtained regarding the volume mean diameter (D_v,50_) and relative width of the distribution (Span) are summarized in Table 1. The water content of the particles was evaluated using Karl Fischer coulometric titration and is also included in Table 1. Both formulations had particles with spherical shapes (although some had smooth shapes and others had some indentations) with a volume mean diameter varying from 2.6 to 2.9 µm, suitable for deep lung deposition, and 11% water content, enough to promote a great and controlled swelling of the particles when in contact with the lung epithelia. 

**Figure 6:**
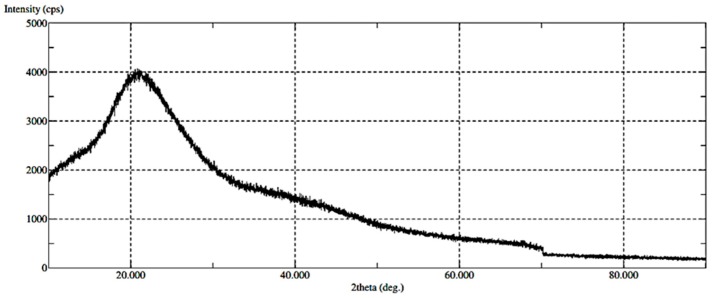
XRD results of the produced CHT_Fe@Au_POX-SH particles.

The presence of amorphous or crystalline states of the materials was investigated through X-ray diffraction (XRD), as demonstrated in Figure 6. The XRD diffractogram identifies the amorphous state of CHT_Fe@Au_POX-SH, similar to the XRD diffractogram reported for CHT alone [32]. No crystalline peaks were observed.

The Fourier Transform Infra-Red (FT-IR) spectra for the Fe@Au_POX-SH and CHT were performed to verify if any new bond was created through the CHT and nanoparticle interaction. The FT-IR spectrum shows no significant changes between both formulations, which can be justified by the large weight ratio of CHT over the nanoparticles.

#### 2.2.2. Aerodynamic Performance

The in vitro aerodynamic performance of all powder formulations was investigated through Andersen Cascade impact measurements, a device that mimics every stage of the respiratory tract from upper airways to alveolus. Also, by determining specific parameters such as emitted fraction (EF), fraction of particles leaving the capsule; fine particle fraction (FPF), the percentage of particles with aerodynamic diameter below 5 µm; mass median aerodynamic diameter (MMAD); and geometric standard deviation (GSD), it is possible to predict particle deposition in the lungs.

**Figure 7:**
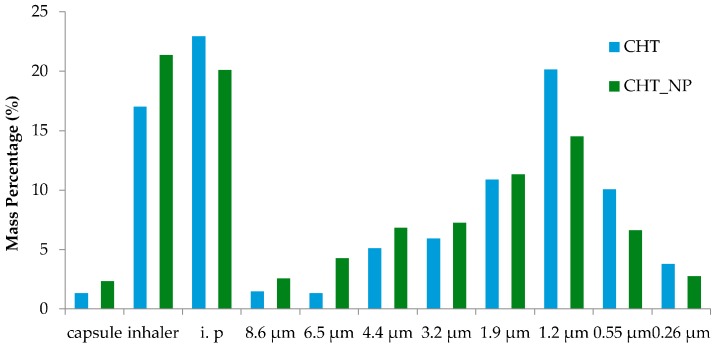
Percentage of mass powder entrapped in the different stages of the ACI. The ACI experiment was performed in a Coppley Scientific instrument at 60 L/min and four kPa. I.p stands for the induction port.

It is well established that the microparticles should have aerodynamic diameters between 0.5 and 5 μm to successfully reach the deep lung. Particles with aerodynamic diameters larger than 5 μm will be trapped in the upper airways and particles with diameters lower than 0.5 μm are exhaled and fail to deposit. Also, particles with sizes below 5 μm may be easily taken up by macrophages [43]. As demonstrated in Table 2, an MMAD of approximately 1.5 μm was obtained and both microparticles, with and without the nanocomposites embedded, reached the final stages of the Anderson Cascade Impactor (ACI), representing the deep area of the lung. By comparing bare chitosan microparticles and the nano-in-micro formulations, it is possible to verify in all the assays that the EF (%) obtained was above 96%, indicating that almost all powder is released from the capsule. In Figure 7, it is possible to perceive that a higher ratio of the powder was collected in the induction port, which simulates the upper airways and the inhaler. This may be due to the formation of turbulent eddies in this zone, which may prevent the aggregated particles from reaching further stages, and to static electricity, respectively [32,44]. It was also found that the FPF (%), i.e., the respirable fraction that is most likely to deposit in the deep lung, was about 55%, exceeding the majority of marketed dry powder inhalers available [45].

#### 2.2.3. Entrapment Efficiency

The entrapment efficiency was calculated to estimate the number of nanoparticles and IBP that remained in the CHT matrix after particle engineering. For microparticles with IBP entrapped and for microparticles with nanoparticles embedded, the entrapment efficiency was 71.7% for IBP and 68.3% for the nanoparticles.

#### 2.2.4. In Vitro Cumulative Studies 

To access particles’ ability to release the entrapped contents, an in vitro study was carried using two different pHs, mimicking the conditions of healthy alveolar epithelial cells (pH 7.4) and cancerous alveolar epithelial cells (pH 6.8). The IBP release mechanism from the nano-in-micro formulations was used to mimic all contents release.

The release profile was adjusted to the Korsmeyer-Peppas mathematical model and the *n*-value was obtained from the slope of the Korsmeyer-Peppas plot and represents different release mechanisms. For spherical geometries: *n* = 0.43 for Fickian diffusion; 0.43 < *n* < 0.85 for anomalous non-Fickian diffusion; *n* = 0.85 for Case-II transport. In both cases herein described, we have obtained a not-Fickian diffusion, since *n* = 0.59 for pH 7.4 and *n* = 0.61 for pH 6.8 (Table 3). Having an anomalous transport, the release rate has the contribution of both diffusion and relaxation mechanisms-stresses during polymer swelling [32,46].

**Figure 8:**
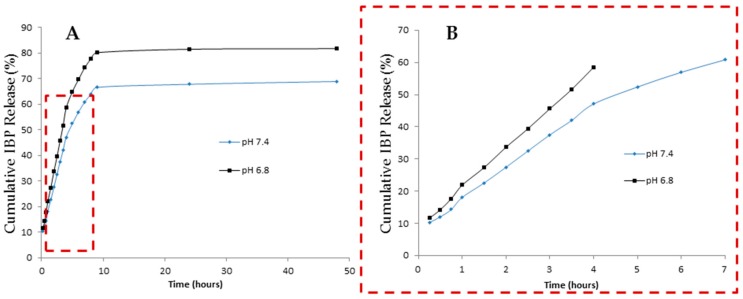
(**A**) In vitro cumulative liberation studies at various pHs; (**B**) Korsmeyer-Peppas adjustment for 60% of the release at different pHs.

As expected, the amount of IBP released from the particles is higher at pH 6.8 than pH 7.4 (Figure 8 and Table 3). Since CHT pKa is around 6.5, at pH 6.8 the CHT amino groups are partially protonated, leading to particle swelling and thus, a faster release. Nevertheless, the sustained and controlled release profile observed at both pHs proves that CHT is a suitable carrier to be used in lung disease situations (such as cancer) through pulmonary administration. After reaching the lung epithelium and upon contact with the diseased cells, the engineered formulations are expected to promote a proper release of the drug or the nanosystems that are entrapped.

## 3. Materials and Methods

### 3.1. Materials

All components were used as received without any further purification. Chitosan (viscosity 5–20 mPa·s) was purchased from Tokyo Chemical Industry (Tamil Nadu, India). The monomer 2-ethyl-2-oxazoline (EtOx, ≥99% purity), boron trifluoride diethyl etherate (BF_3_·OEt_2_), acetone (99.8% purity), acetonitrile (ACN, 99.9% purity), and acetic acid glacial (99.7% purity) were purchased from Sigma-Aldrich (Saint Louis, MO, USA). Ethanol (EtOH) (96% purity) was purchased from Panreac (Barcelona, Spain), Industrial carbon dioxide (CO_2_, purity ≥ 99.93%) was obtained from Air Liquide (Paris, France), and SnakeSkin™ Dialysis Tubing 3500 MWCO (ThermoFisher, Waltham, MA, USA), 22 mm × 35 feet dry diameter, 34 mm dry flat width, 3.7 mL/cm, was purchased from Thermo Fisher Scientific (Waltham, MA, USA).

### 3.2. Synthesis of the Gold Coated Magnetite (Fe@Au)

The synthesis of Fe@Au nanocomposite was obtained using magnetite Fe@SO4^2−^ as the initial support material [25], by polyelectrolyte/gold layer by layer deposition processes developed by Caruso et al. [47]. AuNPs can be adsorbed to form a hybrid nanosystem. In a previous study, Araújo and co-workers proved that by using these layer-by-layer deposition techniques directly on the Fe@SO_4_^2−^, it is possible to obtain a robust Magnetic@gold hybrid with potential applications in biomarker discovery [25].

The synthesis of Fe@Au magnetic nanocomposites was conducted in several simple steps as was already reported by Araújo and co-workers [25], which was performed following a previous procedure for layer-by-layer polyelectrolytes described by Caruso et al. [47,48,49]. PDADMAC and PSS were alternately adsorbed onto the negative surface of the magnetic nanoparticles, Fe@SO_4_^2−^. A solution of 5 mL of the previous Fe@SO_4_^2−^ synthesized NPs was re-suspended in 100 mL of milli-Q water for a duration of 2 min assisted with ultrasonic energy. 100 mL of a water solution containing 1 mg/mL of PDADMAC was added. The solution was maintained in an ultrasonic bath for 1 min, and under magnetic stirring for 2 h. The NPs obtained were separated from the supernatant by magnetic separation and were washed five times with milli-Q water. This process was repeated for the adsorption of the PSS layer, and for the second monolayer of PDADMAC. The three-layer polyelectrolyte magnetite nanoparticles, washed previously, were re-suspended in 100 mL of milli-Q water and were mixed with 5 mL of NaCl 0.2 M solution. This solution was kept for 1 minute in an ultrasonic bath. After this time, 20 mL of previously synthesized gold nanoparticles was added, and the entire solution was maintained with magnetic stirring for 2 h. The final nanoparticles were separated from the supernatant by magnetic decantation, washed five times with milli-Q water, and were finally re-suspended in 100 mL of Milli-Q water. The formation and growth of the partial gold shell was performed based on a previous report [50] reducing aliquots of HAuCl_4_ (100 μL, 5 × 10^−4^ M) with ascorbic acid (100 μL, 0.34 × 10^−3^ M) under ultrasonic stimulation to help nanoparticle separation during the growth process. This gold polymerization process was repeated 15 times. The Strawberry-like nanostructured Fe@Au NPs were separated by magnetic decantation and washed five times with milli-Q water. Subsequently, they were re-suspended in 20 mL of milli-Q water.

### 3.3. Synthesis of the Living Oligooxazoline

The polymerization was carried out following the procedure described by Macedo et al. [51]. Thus, 2-ethyl-2-oxazoline monomer and the initiator BF_3_·OEt_2_ were both added to a stainless-steel reactor, with a monomer/initiator ratio of [M]/[I] = 1/12 [26]. The reaction was carried out under stirring conditions, and the reactor cell was placed in a water bath at 60 °C. Carbon dioxide was introduced into the reactor up to 160 bar. After 24 h of reaction, a continuous washing process was undertaken using CO_2_ for 1 h. Then the pressure was slowly released and viscous foam was obtained inside the reactor.

#### 3.3.1. End-Capping of Living Oligo(2-ethyl-2-oxazoline)

The living Oligo(2-ethyl-2-oxazoline) was end-capped with cysteamine. This process is performed by adding a tenfold excess of cysteamine (POX-SH) (regarding the amount of initiator) solubilized in anhydrous DMF. The mixtures were kept at 70 °C using an oil bath under stirring for 24 h. The oily oligomer solubilized in dry DMF was purified through dialysis against pure milli-Q water. The resulting mixture was dried under vacuum and the resulting oily polymer presented a yellow brownish color. The polymer was soluble in water and shows a blue fluorescence at 384 nm [26].

#### 3.3.2. Nanocomposite Functionalization with OPOX-SH

The Fe@Au NPs were suspended in 10 mL of milli-Q water and were placed in a flask reaction. Under stirring in a US bath, 5 mL of the POX-SH solution in MeOH (5 mg/mL) was added drop-wise. After finishing the addition, the NPs were keep on magnetic stirring overnight. After that time, the nanoparticles were washed in MeOH twice. The final material showed high solubility in MeOH. After repeated magnetic cycles of purification, we observed for the first supernatant a subtle red colour, mainly due to a small percentage of gold nuclei released during functionalization. However, as proven by TEM microscopy (Figure 3), the final nanomaterial exhibits the strawberry structure with the AuNPs anchored to the surface of the magnetic cores.

### 3.4. Nanoparticle Characterization

The size measurements were performed with the end-capped nanoparticles diluted in 1.5 mL of methanol in a Zetasizer Nano ZS instrument (Malvern Instruments, Malvern, UK) in the PROTEOMASS facilities. Zeta potential quantification was carried out in the same Zetasizer Nano ZS instrument using a zeta dip cell. Samples for TEM were prepared by pipetting a drop of the colloidal dispersion onto an ultrathin carbon coated copper grid and allowing the solvent to evaporate. TEM analysis was performed in Spain, at the University of Vigo, CACTI (Center for Researcher and Technical Assistance).

The UV-Vis spectra of the nanoparticles herein developed were acquired using a Perkin Elmer Lambda 25 UV/Vis Spectrometer with a slit width of 5 nm at a scan rate of 240 nm·min^−1^ at 25 °C, in a wavelength range from 350 to 750 nm, and were then analyzed with PerkinElmer UV WinLab™ software (PerkinElmer, Rotterdam, The Netherlands). Fluorescence assays were also performed on a PerkinElmer LS 45 Luminescence Spectrometer with a slit width of 5 nm at a scan rate of 240 nm·min^−1^ using a 10 mm path quartz cell and analyzed using FL WinLab™ software. The excitation wavelength was fixed at 300 nm.

### 3.5. Production and Characterization of the Nano-in-Micro Formulations

#### 3.5.1. SASD Apparatus: Particle Production

The dry powder formulations were prepared by supercritical CO_2_-assisted spray drying. The apparatus is described in detail by Cabral et al. in a previous publication [32]. The SASD is an atomization technology where a solution of the product to be micronized is mixed with scCO_2_ and forced through a nozzle. Therefore, two solutions were prepared: one containing 1% (w/v) CHT in a mixture of 1% acidic water and ethanol (3:2) and the other one containing both 1% (w/v) CHT and the previously synthesized nanoparticles (10:1). In order to test the release profile, a model drug (IBP) was used, adding 100 mg of IBP to the solution of CHT and NPs. CO_2_ is liquefied in a cryogenic bath (−20 °C) and pumped using a liquid pump (HPLC pump K-501, Knauer, Berlin, Germany), which is then heated in an oil bath (70 °C) and sent to the static mixer (model 37-03-075, Kenics-Chemineer, 4.8 mm ID × 191 mm L, 27 helical mixing elements). A high-pressure pump (HPLC 305, Gilson, Middleton, WI, USA) was used to pressurize the solution into the static mixer, allowing the solubilization of the CO_2_ into the liquid solution due to its high surface packing, promoting the near equilibrium. In order to maintain CO_2_ in its supercritical state (above 73.8 bar and 31 °C), the static mixer is enrolled by heating tapes controlled by a Shinko FCS-13A temperature controller (0.2 °C resolution). The pressure in the static mixer is measured using a Setra pressure transducer (0.1 psig stability). The mixture was then sprayed through a 150 µm diameter nozzle into an aluminum precipitator (±0.1 °C), where the primary droplets are formed, and which operates at near-atmospheric conditions. At the same time, a continuously heated air flow enters into the precipitator and dries the particles by evaporating the liquid solvent and promoting the expansion of CO_2_ from the inside of these primary droplets, originating the secondary droplets. The compressed air flow is heated before entering the precipitator. The formed and dried particles exit the precipitator from the bottom side and enter into a high-efficiency (Bucchi) cyclone where they are separated from the gas stream and collected in a glass flask, placed under the cyclone [32,46].

#### 3.5.2. Particle Size Analysis and Morphological Assessment

##### Morphology G3 Analysis

Particle size analysis (sieve and Feret’s diameter) was determined using an optical particle analyzer system (Morphologi G3 Essentials, from Malvern Instruments Ltd., (Malvern, UK)). Both particle size and particle size distributions were measured considering more than 30,000 particles. This characterization was performed in terms of the volume mean diameter (D_v_) and the relative width of the distribution (span). The span is calculated using three measures, D_v,10_, D_v,50_, and D_v,90_ (particle volume diameter corresponding to 10%, 50%, and 90% of the population, respectively) by the following equation:

(1) $$Span=\frac{D_{v,90}-D_{v,10}}{D_{v,50}}$$

##### Scanning Electron Microscopy

Microparticles’ morphology was also determined via SEM (Hitachi, S-2400 instrument; Tokyo, Japan) with an accelerating voltage set to 15 kV and magnifications of 500 and 10 k. All samples were mounted on aluminum stubs using carbon tape and were gold-coated prior to analysis.

##### Fourier Transformed Infrared Spectra

FT-IR spectra were carried out on a PerkinElmer spectrum (Rotterdam, The Netherlands) 1000 FT-IR coupled with Opus Spectroscopy Software with potassium bromide (KBr) tablets containing 20% sample.

##### X-ray Diffraction (XRD)

Samples of CHT_Fe@Au_POX-SH were subjected to XRD analysis (RIGAKU X-ray diffractometer (Ettlingen, Germany), model Miniflex II). Samples were placed in a sample holder and analyzed through CuKα radiation (30 kV/15 mA), with a 2θ angle ranging between 10° and 90° with a scan rate of 1°/min.

##### Karl Fischer Coulometric Titration

The moisture content of each sample was determined using Karl Fischer coulometric titration. 1.5 mg of each sample was placed into the titration vessel and titrated with Karl Fischer reagent, which reacts quantitatively and selectively with water. The instrument was composed of a 831 KF Coulometer and a 728 stirrer both from Metrohm and of a Pt/−20–70 °C electrode.

#### 3.5.3. Aerodynamic Performance

Aerodynamic performance was characterized using an Andersen Cascade Impactor (ACI) (Coopley Scientific, Nottingham, UK). Hydroxypropylmethylcellulose capsules (Aerovaus, USA) were loaded with 20 mg of the dry powder formulation (chitosan with the magnetic nanocomposites).

The total percentage of the powder that is released from the capsule and inhaler, the emitted fraction (EF), was determined gravimetrically and can be expressed as shown in Equation (2):

(2) $$\mathrm{EF}(\%)\;=\frac{m_{full}-m_{empty}}{m_{powder}}\;\times \;100$$

where m_full_ and m_empty_ are the weights (mg) of the capsule before and after simulating the inhalation and m_powder_ is the initial weight (mg) of the powder in the capsule. The respirable fraction (RF) was calculated as the amount of powder reaching the respiratory tract. Fine particle fraction (FPF) was determined by the interpolation of the amount of particles (in percentage) with a diameter below 5 μm emitted from the capsules in each experiment. Mass Median Aerodynamic Diameter (MMAD) was calculated as the particle diameter corresponding to 50% of the cumulative distribution. Geometric standard deviation (GSD) was determined considering the d_84_ and d_16_ measures, which are the diameters corresponding to 84% and 16% of the cumulative distribution, respectively, and can be calculated by the following equation:

(3) $$GSD=\;\sqrt{\frac{d_{84}}{d_{16}}}\;$$

#### 3.5.4. Entrapment Efficiency

The drug encapsulation was determined by milling 20 mg of the produced powder and by then adding a known amount of PBS. The solution was left under stirring for 3 h and was then centrifuged at 15,000 rpm for 7 min. Then, the supernatant was used and the amount of IBP was determined by UV spectroscopy at 225 nm. In order to know the entrapment efficiency of the nanoparticles into the chitosan microparticles, a sample of 20 mg of powder was resuspended in aqua regia and the amount of gold and iron was measured by ICP analysis. The encapsulation (E%) was determined by:

(4) $$E\%=\;\frac{m_{r}}{m_{i}}\;\times \;100$$

where m_r_ is the remaining mass and m_i_ is the initially uploaded mass.

#### 3.5.5. In Vitro Cumulative Release Studies

To evaluate the percentage of IBP that was released from the microparticles, 20 mg of the CHT_Fe@Au_POX-SH_IBP powders were transferred into a snakeskin membrane with a cut off size of 1 μm and were incubated into 5 mL of different pH solutions in a shaking bath (36 rpm and 37 °C): 7.4 and 6.8, pH of an healthy cell and pH of a cancer cell, respectively. At different time intervals, 1 mL of each solution was taken and another mL of fresh solution was replaced. The absorbance from each sample was read using a UV-Vis spectrophotometer at 225 nm.

## 4. Conclusions

In the present work, CHT microparticles containing the functionalized magnetic NPs and IBP were successfully micronized. These powders exhibit appropriate characteristics, either in terms of their size, morphology, and aerodynamic properties, all suitable for inhalation. The most common excipient used in DPIs is lactose (since it is FDA approved) [52] with FPFs ranging from 15% to 45% [53]. PLGA has also been used as a carrier [19], demonstrating high FPF values (30%), however it does not have benefits regarding drug absorption in the lungs. Therefore, CHT with even higher FPF values (approximately 55%) has proven to be a potential carrier when working with pulmonary delivery [53,54]. Also, these values are promising when compared with marketed DPIs, such as the Turbohaler (FPF between 30% and 50%) [55,56].

In this work, CHT has also proven to be a potential candidate to be used as a carrier for therapeutic agents in a lung cancer situation, since it has a higher release percentage (80% release after 10 h) at an acidic pH condition (lung cancer pH).

It is envisaged that fluorescent and magnetic nanoparticles can be used in lung cancer imaging and hyperthermia. However, further studies concerning the use of an applied magnetic field should be performed in order to prove their efficacy.

ACI studies showed an increased fraction of particles reaching the deepest stage of the apparatus. Nevertheless, new experiments need to be performed in order to confirm the statistical significance of the data. To conclude, these proof-of-concept studies suggest that the developed system is a conceivable candidate for pulmonary administration of therapeutic agents for local cancer theragnosis.

## Figures and Tables

**Table 1:** Microparticle composition and properties.

Sample	D_v,50_ (µm)	Span	Water Content (%)
CHT	2.6	0.9	11.1
CHT_Fe@Au_POX-SH	2.9	0.8	11.7

D_v,50_—mean volume diameter; CHT–Chitosan; CHT_Fe@Au_POX-SH–Nano-in-micro formulations of the nanoparticles coated with oligo(2-ethyl-2-oxazoline) end terminated with cysteamine (POx-SH).

**Table 2:** Microparticles’ aerodynamic properties. Comparison between CHT alone and CHT_Fe@Au_POX-SH (CHT_NP), with and without the use of a magnet applied at the last stage of the ACI (Anderson Cascade Impactor).

Assay	MMAD (μm)	RF (%)	FPF (%)	GSD (%)	EF (%)	Yield, ɳ (%)
CHT_NP	1.5 ± 0.1	70.2 ± 0.3	55 ± 2	2.5 ± 0.1	97.50 ± 0.04	38
CHT	1.2 ± 0.2	65 ± 2	48 ± 2	3.4 ± 0.3	96.3 ± 0.1	60

MMAD—Mass Median Aerodynamic Diameter; RF—Respirable Fraction; FPF—Fine Particle Fraction; GSD—Geometric Standard Deviation; EF—Emitted Fraction.

**Table 3:** Peppas’ Adjustment for IBP (Ibuprofen) release.

	*n*	k (h^−1^)	% Mass Released
pH 7.4	0.6	23.1	68.9
pH 6.8	0.6	19.2	81.7

*n*—diffusional exponent; k—constant.

## Abbreviations

The following abbreviations are used in this manuscript:

SASD	Supercritical Assisted Spray Drying
scCO_2_	Supercritical Carbon Dioxide
FT-IR	Fourier Transform Infra Red
TEM	Transmission Electron Microscopy
SEM	Scanning Electron Microscopy
DDS	Drug Delivery System
CHT	Chitosan
NP	Nanoparticles
IBP	Ibuprofen
PDADMAC	Poly-diallyldimethylammonium chloride
PSS	Poly-sodium 4-styrenesulfonate
FPF	Fine Particle Fraction
EF	Emitted Fraction
RF	Respirable Fraction
MMAD	Mass Median Aerodynamic Diameter
GSD	Geometric Standard Deviation
ACI	Anderson Cascade Impactor
